# Links between obesity, weight stigma and learning in adolescence: a qualitative study

**DOI:** 10.1186/s12889-022-12538-w

**Published:** 2022-01-15

**Authors:** Rebecca Langford, Alisha Davies, Laura Howe, Christie Cabral

**Affiliations:** 1 Population Health Sciences, Bristol Medical School, University of Bristol, Oakfield House, Oakfield Grove, Bristol, BS8 2BN UK; 2 Research and Evaluation Division, Public Health Wales, Floor 5, 2 Capital Quarter, Tyndall Street, Cardiff, CF10 4BZ UK; 3 Centre for Academic Primary Care, Bristol Medical School, University of Bristol, Canynge Hall, 39 Whatley Rd, Bristol, BS8 2PS UK

**Keywords:** Child, Adolescent, Obesity, Weight stigma, Education, Schools, Qualitative, ALSPAC

## Abstract

**Background:**

Educational attainment is a key social determinant of health. Health and education are linked by multiple pathways, many of which are not well understood. One such pathway is the association between being above a healthy weight and lower academic achievement. While various explanations have been put forward to explain this relationship, evidence for causal pathways is sparse and unclear. This study addresses that evidence gap.

**Methods:**

We interviewed 19 adults (late 20s; 14 female, 5 male) and one young person (14 years, male) from the UK in 2019/2020. Participants were recruited from the ALSPAC 1990s birth cohort, sampled to ensure diversity in socio-economic status and educational attainment, and a community-based weight management group for young people. Interviews focused on experiences of being above a healthy weight during secondary school and how this may have affected their learning and achievement. Interviews were face-to-face, digitally recorded, and transcribed verbatim. We analysed the data thematically.

**Results:**

We identified key pathways through which higher body weight may negatively impact educational performance and showed how these are linked within a novel theoretical model. Because larger body size is highly stigmatised, participants engaged in different strategies to minimise their exposure to negative attention. Participants sought to increase their social acceptance or become less socially visible (or a combination of both). A minority navigated this successfully; they often had many friends (or the ‘right’ friends), experienced little or no bullying at school and weight appeared to have little effect on their achievement at school. For most however, the behaviours resulting from these strategies (e.g. disruptive behaviour, truanting, not working hard) or the physical, social or mental impacts of their school experiences (e.g. hungry, tired, self-conscious, depressed) made it difficult to concentrate and/or participate in class, which in turn affected how teachers viewed them.

**Conclusions:**

Action to combat weight stigma, both within schools and in wider society, is urgently required to help address these educational disparities that in turn can impact health in later life.

**Supplementary Information:**

The online version contains supplementary material available at 10.1186/s12889-022-12538-w.

## Introduction

The relationship between education and health is synergistic. Educational attainment is a key social determinant of long-term health outcomes [1–3] and a predictor of adult obesity [4]. In turn, health status, particularly during adolescence, has an impact on educational attainment and lifetime social outcomes [5]. The multiple causal pathways linking education and health are complex and many gaps remain in this evidence base [5–7]. One such gap is the association between being above a healthy weight and lower academic achievement [8–11]. Various direct and indirect explanations have been posed to explain the relationship between body weight and academic achievement, including health-related absences [12], differences in cognitive processes [13], the impact of weight-related bullying [14], and the unconscious bias of teachers [15]. However, empirical evidence to support these hypotheses is scarce and inconsistent, making it hard to draw clear conclusions [16, 17].

The pathways linking body size and academic achievement are likely to be highly complex and socially constructed. There have been surprisingly few qualitative studies which ask young people their views on this issue. Most focused on the psychosocial impact of having a larger body size or their experience of treatment for their ‘condition’ (see [18]). Of those conducted within educational settings, just three examined how weight might affect educational performance. Martin et al. [9] found teenage girls with obesity felt they spent more time on schoolwork due to the absence of friends. Brown [19] described how the physical discomfort felt by American undergraduates using chairs and desks that were too small for them made them feel unwelcome in the classroom. Kenney et al. [20] reported teachers felt students with obesity were more likely to struggle at school, with low self-esteem and weight-related bullying believed to reduce participation in class. Our current understanding of how weight status impacts on learning is therefore limited.

This study sought to build on and expand the findings of earlier literature by using in-depth interviews to explore pathways through which weight may affect learning in secondary school. We approached this project viewing obesity as resulting from the interplay between genetic predisposition and exposure to obesogenic environments [21]. However, obesity is a highly stigmatised condition and weight stigma is a stressor which can promote weight gain [22, 23]. Adolescents may be particularly vulnerable to the negative social and emotional consequences of weight stigma [24]. Understanding these experiences and how young people react to them may help us better understand the link between weight and academic achievement during adolescence, and importantly, inform approaches to ensure all children achieve their educational potential.

## Methods

### Population, recruitment and sampling

We recruited adult participants from the Avon Longitudinal Study of Parents and Children (ALSPAC). This is a UK population-based birth cohort which recruited 14,541 pregnant women from Bristol and the surrounding areas between April 1991 to December 1992 [25–27]. Biological and behavioural data have been collected from this cohort from before birth to early adulthood; participants were 27–28 years at time of recruitment. A link between higher Body Mass Index (BMI) and lower academic attainment has been established in this cohort [28]. (Additional cohort details in supplementary material).

We emailed participants inviting those who self-identified as being above a healthy weight during secondary school to take part in an interview. We contacted interested participants by phone/email to answer questions and collect brief background data to allow sampling of participants from diverse socio-economic situations and with a range of educational attainments based on self-reported grades.

In addition, we attempted to recruit young people who were currently above a healthy weight via community-based weight management services for young people (11–16 years). Working with services in two local authority areas (Bristol and South Gloucestershire), we invited current participants of weight-management groups to take part. Only one young person (14 year-old male, “Tim”) agreed to be interviewed.

Sample size was not pre-determined allowing data collection to continue as new ideas were identified [29]. By the final interviews (participants 16–20) we saw consistency in content with previous interviews with no new substantive themes identified. We were unable to interview two further participants who had expressed interest; all data collection with the ALSPAC cohort was suspended in March 2020 due to the Covid-19 pandemic.

### Data collection

Participants were interviewed by RL (lead researcher) face-to-face between July 2019-Feb 2020. Interviews focused on participants’ experiences at school, feelings about their size, and if/how their weight affected learning at school. We developed a topic guide to facilitate a sensitive and flexible approach to interviewing (see supplementary materials). We adapted our methods for adult and young person interviews, recognising the differences in focus: retrospective reflections or descriptions of current lived experience. Retrospective ‘life history’ interviews [30] proved useful with adult participants, allowing them to reflect on the links between weight and their educational experience as a whole. To facilitate this, we constructed a timeline with participants during the interview allowing them to identifying key moments in their educational career (e.g., moving to a new school, performance in exams, periods of bullying) while documenting changes in their weight over the same period. The topic guide for the adolescent participant did not use a timeline and focused on experiences since starting secondary school. Interviews were usually an hour long, digitally recorded, and transcribed verbatim. Audio-files, consent forms, anonymised transcripts and timelines were stored securely. We reimbursed participant travel costs and provided a £20 shopping voucher as thanks. Participant names are pseudonyms.

### Data analysis

**Fig. 1:**
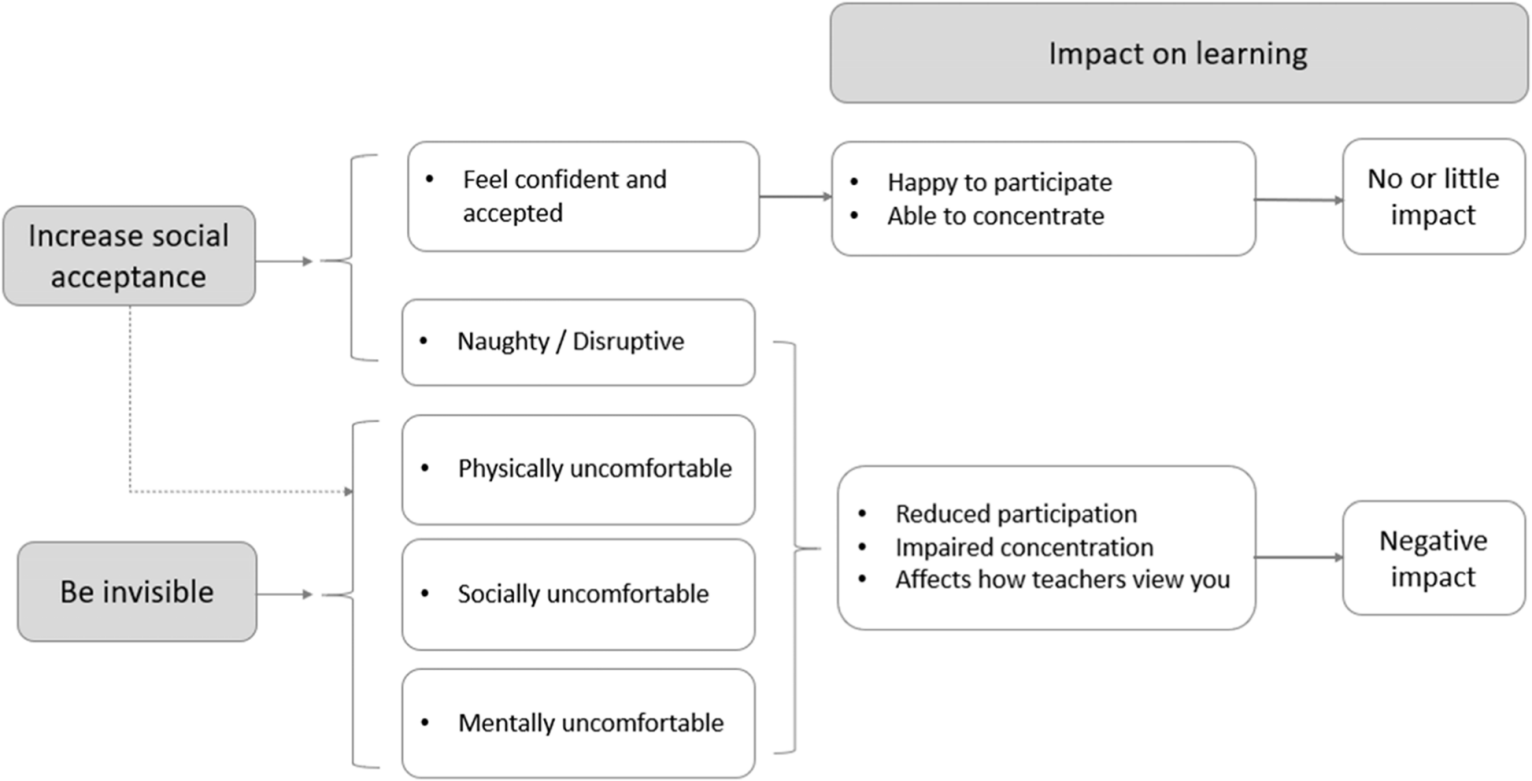
How “survival strategies” adopted by students with larger bodies can affect learning. Theoretical model showing how different “survival strategies” can impact on feelings and behaviours and potentially impact on learning. The two strategies are not mutually exclusive: participants could seek to increase social acceptance while still experiencing discomfort at school (indicated by dotted line)

Transcripts were analysed using a thematic approach [31]. Transcripts were read by RL (lead researcher) and CC (member of the study team) to familiarise themselves with the data, independently noting potential codes. Through discussion we developed these into a coding framework which was subsequently applied to further transcripts by RL. We made additions and modifications to this framework as necessary, meeting regularly to discuss coding and development of our analysis. Codes were grouped together, split into sub-codes, subsumed into higher-level concepts and ordered hierarchically to eventually create overarching themes and sub-themes. We explored relationships between codes/themes and potential causal pathways by visually ‘mapping’ these concepts. This map was revised iteratively as analysis progressed to create our final theoretical model (Fig. 1). We used NVivo12™ to facilitate data management and interpretation.

### Public involvement

We consulted a young people’s advisory group (https://arc-w.nihr.ac.uk/news/power-to-the-young-people/) and local secondary school teachers to advise on recruitment approaches, appropriate methods, and the study focus. Following this advice, we use terms such as “larger bodies” or “above a healthy weight” when discussing our data and “obese” and “overweight” when citing published literature. We discussed our findings with staff (*n* = 40+) at a local school conference and with another young people’s advisory group (17 participants, aged 16–21 years: https://decipher.uk.net/public-health-improvement-research-networks-phirns/public-involvement-alpha/). These were conducted online due to Covid-19 restrictions.

## Results

We interviewed 19 adults (27–28 years) and one young person (14 years) in total (14 females, 6 males). Table 1 provides a summary of participants’ educational achievement and socio-economic status.

**Table 1:** Participant characteristics

Gender	*Female*	14
	*Male*	6
Educational achievement	*High*	6
	*Medium*	7
	*Low*	7
Socio-economic status	*High*	6
	*Medium*	7
	*Low*	6
	*Unavailable*	1

Educational achievement based on GCSE scores (C grade = pass): ‘high’ = mostly A/B grades; ‘medium’ = mostly C grades; ‘low’ = mostly D/E grades. Socioeconomic status measured by proxy variable of maternal education

Through our analysis we constructed three key themes. The first two – “increase social acceptance” and “be invisible” – represent "survival strategies" used by participants to navigate the complex social world as a student with a larger body. The third theme focuses on the impact of these strategies on their ability to learn. From these themes we constructed a theoretical model to explain the way(s) in which body size may affect learning (Fig. 1).

### Theme 1: increase social acceptance

Social acceptance and friendships were key to navigating the school social world. *“Loners”* with larger body sizes were an *“easy target”.* Friends offered protection against bullying or teasing. The wider your friendship circle or the more popular your friends, the greater the protection offered against weight-based teasing. As Rachel explained, people rarely bullied her *“because I was probably friends with one of their friends… I was kind of in a little safe zone.”*

Participants appeared to cultivate certain social identities to enhance social acceptance. Some of these identities worked well, increasing people’s acceptance and confidence, and potentially benefiting (or not harming) their ability to learn. However, more commonly these identities were associated with behaviours likely to inhibit learning.

#### “Naughty”

Several participants described themselves as one of the *“naughty kids”* who messed about to make people laugh. Suze explained she was *“quite naughty… the class clown”* and had *“no respect for teachers”.* She speculated this behaviour was to gain social acceptance: *“I don’t know if that was trying to make people like me maybe… to get attention from the other kids?”* Jack linked his class clown role to his insecurity around his weight: *“In hindsight [I] probably was doing it to fit in, to impress.”* Tim, the 14 year-old participant, was more matter of fact: *“You want to be seen as a cool person so you’d do bad things, get sent to isolation*”.

For Kerri, her disruptive behaviour was about finding a place to fit in. To be “in” with the popular girls *“you have to be really skinny and pretty”.* Her exclusion from this group pushed her towards the *“naughty kids”* who accepted her: “*You feel like you don’t fit in so then you turn to be a bit mischievous and stuff. You get in with those sorts of people. They’re like the same.”*

#### “Popular”

Gareth recognised being the *“jokey kid”* would make people like him, making him less of a target for bullying. As he explained, *“if I’m not going to be one of the good-looking kids because of my size, then maybe being someone with a nice personality will help me make friends?”* His social acceptance was cemented later in school when his size made him a valued rugby player shifting him *“a bit more up the social ladder”* and widening his friendship circle to include *“all the cool jock kids”.*

Sean benefitted from friendship with two of the most popular boys in school, former primary-school friends. This association meant he was *“saved from the brunt” of* weight-based teasing: “If someone did make a comment straight away, one of my other friends would’ve been like, “F*** off. Don’t talk to him like that….”

#### “Nerdy”

In some cases, constructed identities positively impacted on learning. Isobel was acutely insecure about her weight at her private school. Struggling initially to make friends, she developed a bookish personality:

*I wasn’t able to control my weight but I can control this idea of making myself do lots of work… I definitely put a lot of energy … into making sure I would be seen in teachers’ eyes as someone who was academically able.*

Amanda described herself as “*a bit nerdy.”* In her private school context, her academic achievement was a valuable social identity, giving her a confidence which she described as a *“shield”* against negative attention. State school student Hannah used her status as a “bright” student to create links with other people: *“I was able to help my friends and people that weren’t necessarily my friends… they could come to me and I could help*”.

#### “Thin”

In some cases, creating a valued identity was not about developing a more acceptable personality, but simply attaining the ideal “slim” body. Around 15/16 years Alexa became unhealthily obsessed with *“major calorie counting”,* rapidly losing weight over a summer holiday. Where previously she had felt *“segregated”* because of her size, her new slim body brought a change: *“[When I] lost the weight…that bullying went away. I definitely can see there is a link.”*

Similar stories were reported by other female participants. Isobel was deeply unhappy *“being the fat one”* and *“starved” herself to lose weight.* Likewise, Suze and Emma lost weight to fit in with others and increase their self-confidence. Amy naturally became slimmer in her final school years and noted the social benefits of her new slim body. Whereas before she was badly bullied, “*in Year 11 people actually started to come round and actually speak to me. That I entirely put down to the fact that I [had] a smaller body”.*

### Theme 2: be invisible

For participants who struggled to forge friendships and gain social acceptance, an alternative strategy was to become as invisible as possible at school. Many participants felt rejected and bullied because of their weight. Consequently, they tried to avoid attention. Though Gareth later gained popularity by being the “jokey” one, initially he tried to *“fly under the radar”* explaining, *“you’re just the short fat kid… keep your head down… There’s no point sticking out.”* Similarly, Amy described gravitating to people who did not stand out: *“I put myself automatically with the people that didn’t get noticed.”*

However, the strategies used to become “invisible” or the consequences of failing to remain inconspicuous often resulted in participants feeling deeply uncomfortable at school.

#### Physically uncomfortable

Several participants described never eating at school for fear of comments or teasing, resulting in persistent feelings of hunger or lethargy. As Matt explained “*I was really worried that if I was eating food, people would be like, “Oh my God, he’s that big and he’s eating food.“* Other participants talked about physical discomfort related to clothing. Some wore loose, baggy layers to disguise their bodies which made them deeply uncomfortable during warmer months: *“Even during heat waves I would never take my jumper off. I was ill from it because I would just never want people to see my body”* (Amy).

#### Socially uncomfortable

Most participants were extremely self-consciousness of their appearance. They described feeling *“insecure”, “lacking confidence”* and being acutely aware they were *“different”.* This social uncomfortableness could dominate their everyday experience of school. Sarah felt deeply isolated because of her weight: *“just being very self-conscious with no confidence and… every day going in and being on my own*”. Rachel described a pervasive anxiety that attention might fall on her at any moment:

*Even if you’re just sat in assembly … They’re chatting about the top male rugby players but you’re still like, “What if they say my name, then attention is going to be drawn to me?” You’re that worried about it.*

#### Mentally uncomfortable

Many participants described being left out, teased or bullied about their weight throughout school. Many also described feeling unhappy about their body size. However, for some participants their experience went beyond discomfort and unhappiness into what they described as depression and/or anxiety. Lauren described the link between her mental health and weight and how this exacerbated her isolation in school: *“Anxiety, depression and my weight have followed me all the way through my life… I just became very withdrawn found it hard to communicate with people… I didn’t know what to do with myself”.*

In some cases, participants started to skip classes. Kerri explained she truanted because *“I felt quite depressed at times. I would actually walk… out of school and go home*”. Kirsty similarly truanted, initially because she was so unhappy at school, but later to avoid further bullying: “*all my other friends were doing it then… I thought the bullying would start again, so I just followed them”.*

### Theme 3: impact on learning

A minority of participants appeared to successfully navigate school with no perceived detriment to learning. Amanda, Sean and Gareth were all confident, had good friends and largely avoided any weight-based bullying. Consequently, weight had little impact on their behaviour or performance at school. As Amanda concluded, *“I was very well-behaved, I took part in class, had a good relationship with my teachers and I felt confident.”* In another case, body size potentially improved learning and achievement. Though Isobel lacked social confidence, her insecurities also motivated her to apply herself: *“[my weight] made me more insecure in school, but in a way it pushed me to be like, ‘I’m going to work harder.’”*

However, most participant narratives suggested a detrimental impact on learning, through reduced participation and concentration in class or how teachers viewed them.

#### Participation

Participants who tried to be “invisible” at school would rarely participate in class. They talked about being *“worried,” “anxious,”* and even *“terrified”* at having to speak in class. They seldom answered questions or volunteered and were reluctant to seek help from teachers for fear of drawing negative attention.

*I think a lot of it came from being in class and not wanting to be asked questions and looked at. That made me worried, it made me feel uncomfortable, and just not want to be there.* (Emma)

Depression could also affect classroom participation. Lauren felt unable to participate because *“you just don’t have any give in you anymore to be an active member of the class.”* Overall, the common picture described by these participants was of a passive classroom experience. As Kirsty described, *“I just sat there and listened and wrote in my book. I never really took part”*.

#### Concentration

Participants described multiple ways in which their concentration was impaired. For some, it was as basic as being too hungry or tired to concentrate having skipped meals at school. Several talked about *“zoning out”* or *“drifting off”* in class: *“I would be falling asleep… in lessons I wasn’t focused because I hadn’t eaten anything”* (Matt).

Participants who identified as *“naughty”* described a lack of attention in class. Jack felt insecurities about his weight led him to mess about in class to make others like him, explaining, *“I was always quite socially conscious within the classroom and never really particularly focused*”. Suze similarly played up in class to impress friends, explaining she would *“just be really bad…. not listen at all, just basically talk over [the teacher] or not sit and work.”*

Other participants linked poor mental health to impaired concentration. Alexa and Isobel both described persistent feelings of weight related social anxiety at school making it hard to focus on anything else: *“You’re just constantly thinking that you don’t want to be that weight”* (Alexa). Amy’s depression led to suicidal thoughts which meant she couldn’t focus in class: *“I would just think about [suicide] all the time.”* Lauren noted the profound effect depression had on her ability to learn:

*I wasn’t concentrating… I wasn’t really paying attention. Because I was depressed about the way I looked nothing would go in… My brain physically couldn’t comprehend things because there was so much in there already.*

#### How teachers view you

Some participants felt they were unfairly labelled as “troublemakers” by teachers. Matt was disciplined for swearing at a teacher and refusing to take part in PE lessons. Exposing his body while getting changed was traumatic and a key trigger for bullying (of which his teachers were unaware). Tim (14 year-old) had purposely been naughty in class the previous year to impress others, but was now working harder in school. However, he felt his teachers had failed to acknowledge his improved efforts and continued to view him as naughty *“because of last year [and] how I acted.”*

Some felt teachers simply saw them as shy, quiet students. Some teachers tried to get students to speak in class, which participants often found a deeply uncomfortable experience. Others seemed to realise how difficult this was and left them alone. However, as Rachel suggests, being “invisible” in class meant teachers were perhaps unaware of students’ true potential.

*Half the time they probably didn’t even know you knew the answer. They just think people are shy and just need to get over it. They don’t think, “Why is that person shy?” They don’t really look.*

### Did participants feel their weight affected their learning?

Most participants saw a clear link between how they felt about their bodies, their behaviour and experiences in school, and their ability to learn. Several participants felt they would have been more confident and more willing to take part in class had they been thinner in school. Others suggested they would have been less likely to mess about in class. Jack felt he might have been *“less of a prat”* in class if he had felt more confident, while Kerri believed *“if I was skinny I would be with the pretty girls wh[o] were never naughty.”* Kirsty was aware she would have engaged more and truanted less if not bullied: *“I would’ve participated better. I would’ve wanted to be in that class, I would’ve known a lot more than skipping it.”*

Overall, many participants felt they would have done much better at school under different circumstances. Importantly, this was not just expressed by those who did less well at school, but also by those with good grades but who felt they had not fulfilled their potential:

*I passed, but I could have easily got a distinction… I could have probably done better if I had focused more but…learning was really difficult because I was in an environment I didn’t want to be in all the time.* (Amy)

## Discussion

Our study is one of the first to explicitly explore the relationship between body size/weight and educational attainment drawing on lived experience. We constructed a novel, theoretically informed model illustrating potential causal pathways. To understand our results, we drew on Major & O’Brien’s [32] Identity Threat Model of Stigma. This model suggests individuals become aware they possess a stigmatising attribute and consequently belong to a devalued group. This awareness results in a threat to their identity when the demands imposed by a stigma-relevant stressor (e.g. large body size) are viewed as a) potentially harmful to one’s social identity and b) exceeding one’s personal resources to cope with those demands ([32]:p399). Major & O’Brien link coping mechanisms with the body’s fight or flight instincts, characterising them as either “engagement” or “disengagement” strategies. Importantly, this model emphasises the variability in responses to stigma, noting that “high status” and “low status” groups will interpret and react very differently to being the target of a stigmatised stereotype.

Viewed through this lens, the behaviours and experiences described by our participants make sense. All participants were aware their weight made them vulnerable to negative attention – a potential threat to their identity. Some participants “engaged” with this threat, mitigating it by actively seeking to create social acceptance in other ways: by being “popular” or “naughty”, developing a “nerdy” identity or dramatically losing weight. Others, lacking the resources to create social value in other ways, attempted to avoid this threat by becoming inconspicuous and “invisible” as far as possible.

As predicted by the Identity Threat Model, the impact of weight stigma on learning was not universal. A minority of participants did not appear to experience negative impacts on their learning. These participants were “popular” or well-liked for some reason, giving them higher status. In this context, though aware of the stigma attached to their weight, this threat did not exceed their resources to cope with this demand. For others, the impact on their learning was more significant as their behaviours or negative experiences impacted on their ability and willingness to participate and concentrate in class. Once again, this fits with Major & O’Brien’s [32] theory, which suggests strategies employed to achieve one goal (e.g. preserve self-esteem) may hinder the achievement of others (e.g. academic performance).

### Links to wider literature

Many of our participants reported a lack of engagement in classroom activities which may have impaired their learning. Student engagement comprises behavioural, emotional and cognitive elements [33] and is commonly viewed as essential to successful learning. A recent meta-analysis found engagement to be an important predictor of academic achievement [34]. The largest effect was found for the “behavioural” dimension suggesting that conduct, effort and participation in class may be driving this association. Corroborating our qualitative findings, a recent study by Finn and colleagues [16] found classroom participation was lower for students with higher BMIs and concluded this mediated the relationship between weight and academic attainment.

Difficulty concentrating in class was also raised by many of our participants, often linked to skipping meals or poor mental health. Though skipping breakfast has been associated with poorer cognitive or educational outcomes [35], the impact of skipping lunch is less clear [36]. Nonetheless, several participants discussed being distracted by feelings of hunger or lethargy in class. A minority of participants also described impairing levels of anxiety and depression during secondary school, often related to their weight. Several studies have found people with obesity are more likely to experience depression [37, 38] and anxiety [39] and these experiences may plausibly inhibit academic attainment [40].

Our findings complement Kenney et al.’s [20] study on teacher perspectives of students with obesity. Teachers suggested these students often fell into stereotypical personality types. Most were characterised as shy, reserved and reluctant to participate, congruent with our “be invisible” theme. However, teachers also suggested they could be “class clowns” or bullies. While some of our participants identified as clowns, unsurprisingly none identified themselves as bullies. Nonetheless, such behaviour is compatible with our theoretical model as a means of creating social acceptance by picking on weaker peers.

Kenney et al. (2017) also suggested a minority of teachers held negative views about students with obesity and treated them unfairly. We found little evidence of unfair treatment in our participants’ narratives, but several suggested teachers underestimated their true potential, mistaking their lack of participation as shyness or lack of knowledge or interest. Other studies showing students with obesity are perceived as less capable [15] or are given lower grades by teachers [41] suggests unconscious teacher bias may be a route through which academic achievement is impaired.

### Implications

Action is needed on multiple levels to encourage students with larger bodies to participate in class and improve educational attainment. First, schools should adopt a zero-tolerance approach to weight-based bullying with explicit reference to this in anti-bullying policies and practices. A cross-national study identified “being fat” as the most likely reason for youth bullying, far ahead of other characteristics like ethnicity or sexuality [42]. Yet a review of 10 systematic reviews and meta-analyses found none of the 275 included interventions explicitly addressed weight-related bullying [43], perhaps reflecting that weight stigma remains one of the last acceptable forms of discrimination [44].

Second, research is needed on how best to address weight stigma within educational settings. School-based interventions can increase personal body satisfaction [45], but research on reducing weight bias in school settings is scarce. A review by Daníelsdóttir et al. [46] identified only two weight-bias reduction interventions targeting adolescents (with mixed effects), while Nutter et al. [47] identified five interventions with pre- or in-service teachers (with largely positive effects). Further development, co-production and evaluation of such interventions is urgently required.

Finally, work remains to be done at a societal level to address weight bias. Beliefs that obesity is the result of unhealthy behaviours and reversible through personal effort are widespread [48], despite extensive evidence for the genetic, environmental and commercial determinants of obesity [21, 49]. Those who view obesity as a matter of personal responsibility are also more likely to hold weight-stigmatising views [50]. The media plays a part here: Flint and colleagues found “unequivocal evidence” of weight stigmatization in UK national newspapers [51]. Even public health obesity campaigns have been accused of increasing weight stigma [52–54]. Interventions to address the determinants of obesity must avoid inadvertently increasing weight stigma, particularly when targeting young people.

### Limitations

Participation was based on self-identification as being “above a healthy weight” during secondary school, rather than BMI measurements. Most of our interviews (19/20) were with adults and offer a retrospective ‘life history’ account of their secondary school experiences. Such interviews offer rich understandings of the experiences at a certain point in life but may be subject to recall bias and post-hoc interpretation. We also sought to recruit young people (11–16 years) to explore current lived experiences but were only able to recruit one young person. While this young person’s narrative raised similar issues to our adult participants, it presented current lived experience rather than a reflection on past events. Additional interviews with young people would have strengthened the study.

We discussed our findings with young people and teachers in two on-line discussion groups. Both groups felt our findings were largely congruent with their own experiences. Social media and cyber-bullying were raised in both groups, neither of which were prevalent when most of our participants were at school in the 1990s. These issues warrant further investigation as to how they add to or change our theory.

The interviewer (RL) is a slim white woman. She was mindful of the “slim privilege” [55] this afforded her in conducting this study. It is possible her weight status affected the data collected during these interviews. However, she developed good rapport with participants and conducted interviews with sensitivity and respect.

## Conclusion

A growing body of evidence suggests an important link between higher body weight and reduced academic attainment which in turn influences lifetime health outcomes. Our research elucidates the pathways through which weight stigma may impact behaviours in school and thus potentially affect learning and educational achievement. Action to combat weight stigma, both within schools and in wider society, is urgently required.

## Supplementary Information

**Additional file 1.**

**Additional file 2.**

## Data Availability

The datasets used and/or analysed during the current study available from the corresponding author on reasonable request. This request will be subject to the approval of ALSPAC and the Data Access Committee at the University of Bristol. Restrictions apply to the availability of these data, which were used under licence for this study.

## Abbreviations

ALSPAC: Avon Longitudinal Study of Parents and Children
BMI: Body Mass Index
UK: United Kingdom
