# Supplementary material for: Links between obesity, weight stigma and learning in adolescence: a qualitative study
Source: BMC Public Health. 2022 Jan 15;22:109. doi: 10.1186/s12889-022-12538-w (PMC8761050; doi:10.1186/s12889-022-12538-w)
Supplement: Supplementary file 2 — Additional file 2. [file 12889_2022_12538_MOESM2_ESM.docx]

**
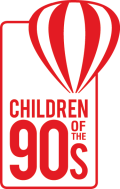
EVERYBODY LEARNING (ALSPAC) TOPIC GUIDE**

## Before starting the interview

Ensure participant has read information sheet and consent form has been signed. Remind participants: the interview will be recorded; they can skip questions or pause/stop the interview at any time; the interview is confidential (unless safeguarding issue arises).

## Preamble

We’re interested in how and why body size might affect young people’s experiences at school/college/uni and how they learn. It’s not a test, there are no right or wrong answers. We’re interested to hear your thoughts on this.

## Timeline

Go through timeline with participant to mark out their educational experience (school, further education, higher education, jobs etc).

## School experience

| - Tell me about what secondary school was like for you… | - *How did you feel about it?* - *Liked/disliked it – what and why?* - *Happy there? Why/why not?* |
| --- | --- |
| - How did you do at school academically? | - *Where do you think were are on a scale? (bright/brainy middling, struggling)* - *In different subjects?* - *Why do you think that is?* - *How did you do in your exams?* |
| - How did you do at school socially? | - *Did you have many friends?* - *Did you fit in at school?* - *Any trouble with students/ teachers?* - *Why? Tell me more?* |

## Further/Higher/Vocational education

Ask these question if relevant (refer to timeline)

| - What about after school? Tell me about your experiences at college/vocational training/university etc | - what and where they studied? - how they did academically? - how they did socially? - better or worse than school? Why? |
| --- | --- |

## Body size

| - Refer back to timeline – how would you describe your body size at different periods in your life? | - *What language are you comfortable with?* - *Why do you think you were that size then?* - *Why did it change (if it did)?* |
| --- | --- |
| - How did you feel about your weight or body size back then? (identity) | - *Did it affect how you felt about yourself?* - *Why is that? Tell me more* - *How do you feel about it now?* |
| - Did your weight ever affect how people treated you? | - *Friends, family, strangers* - *Examples? Why?* - *Tell me more about that* |
| - What about at school/college/uni/training? Did your weight affect you there? | - *Examples? Why?* - *Tell me more about that* - *Difference between school/college/HE/workplace?* |

## Learning (using card prompts)

These are some of the things we think affect how people learn and how well they do school. We’re interested in whether people’s body size and weight might affect any of these.

| - Do you think your weight ever affects any of these? Pick out the top 3-4 issues | |
| --- | --- |
| - Tell me why you picked those cards? | |
| - How did your weight affect [topic picked]?   - Can you give me any examples?   - Why did it happen?   - How did it make you feel?   - Did this affect just you or other students who are above a healthy weight too? (gender)   - Did it have more of an effect at some times than others?   - Was it different in college/university/vocational training? | |
| - How did it affect your learning? | - - In class, taking it in/remembering, homework, tests/exams, relationship with teachers   - Did it affect how well you do e.g. grades/tests? |
| - Is there anything that could have be done to change this or make it better? | - *What do you wish you could change?* - *Probe: responsibility (themselves, other students, teachers/school)* |
| - What about the other cards you didn’t pick? | - *Any other relevant ones?* - *Tell me why/why not* |
| - Is there anything missing here, that we haven’t mentioned? | |

## Employment & the workplace

We’re also interested in if body size and weight affect people as they enter the workplace. (Only ask if relevant).

| - Tell me a bit about what jobs you’ve done since leaving education… | - *Refer to timeline* |
| --- | --- |
| - Do you think your weight has ever affected your employment? | - *Qualifications affecting employment* - *Getting a job (interviews, confidence)* - *Mental/physical problems* - *How treated by others* - *Promotion and opportunities* |

*Close*

| - What’s the most important thing we’ve discussed today? |
| --- |
| - Is there anything else you want to tell me? Do you have any questions for me? |

## Thank them for their participation

Card topics will include: concentration, confidence, behaviour, not taking part in lessons, getting into trouble, bullying, mental health and anxiety, relationship with students, relationship with teachers, attendance, enjoying school, exercise, food/hunger, sleep, being treated fairly, physical discomfort

**EVERYBODY LEARNING (YOUNG PERSON) TOPIC GUIDE**

## Before starting the interview

Ensure participant(s) have read information sheet and consent/assent forms have been signed. Remind participants: the interview will be recorded; they can skip questions or pause/stop the interview at any time; the interview is confidential (unless safeguarding issue arises). If paired interview, set ground rules around not discussing what is said with others.

## Preamble

We’re interested in how and why body size might affect young people’s experiences at school, how they learn and how well they do at school. We’re talking to you because you’ve taken part in the Alive N Kicking or REACH course. It’s not a test, there are no right or wrong answers. We’re interested to hear your thoughts on this.

## School experience

| - Tell me about the school that you go to… | - *What’s it called? Includes sixth form? Size? Good school? Have you changed schools? What year are you about to go into?* |
| --- | --- |
| - How do you feel about school? | - *Like/dislike it – what and why?* - *Happy there? Why/why not?* |
| - How do you think you’re doing at school academically? | - *Where do you think you are on a scale (bright/brainy middling, struggling)* - *In different subjects?* - *Why do you think that is?* - *How did you do in your exams?* |
| - How do you think you’re doing at school socially? | - *Do you have many friends?* - *Do you fit in at school?* - *Any trouble with students/ teachers?* - *Why? Tell me more?* |

## Body size

| - Is it Ok to talk a bit about your weight now? | |
| --- | --- |
| - How did you get involved in REACH/AnK? | - *How referred in? Was it useful?* |
| - How would you describe your weight or body size? | - *Do you know your BMI?* - *What language are you comfortable with?* |
| - How do you feel about your weight or body size? | - *Does it affect how you feel about yourself?* - *Do you think about it much?* - *Why is that? Tell me more* |
| - How long have you been above a healthy wgt? | - *Why do you think that is?* |
| - Do you think your weight ever affects how people treat you? | - *Friends, family, strangers* - *Examples? Why?* - *Tell me more about that* |
| - What about at school? Does your weight affect you there? | - *Examples? Why?* - *Tell me more about that* |

## Learning (using card prompts)

These are some of the things we think affect how people learn and how well they do school. We’re interested in whether people’s body size and weight might affect any of these.

| - Do you think your weight ever affects any of these? Pick out the top 3-4 issues | |
| --- | --- |
| - Tell me why you picked those cards? | |
| - How does your weight affect [topic picked]?   - Tell me a bit about what happens   - Why does it happen?   - How does it make you feel?   - Are some lessons/teachers better than others? Why?   - Have you always felt like this or has it got better/worse? Why?   - Does this affect just you or other students who are above a healthy weight too? (gender)   - Do your teachers/other students/friends notice what’s happening? - How does it affect your learning? (in class, taking it in/remembering, homework, tests/exams, relationship with teachers)   - Does it affect how well you do e.g. grades/tests? | |
| - Is there anything that could be done to change this or make it better? | - *What do you wish you could change?* - *Probe: responsibility (themselves, other students, teachers/school)* |
| - What about the other cards you didn’t pick? | - *Tell me why/why not* |
| - Is there anything missing here, that we haven’t mentioned? | |

*Close*

| - If you could tell your school, teachers or other students one thing about topic, what would it be? | - What’s the most important thing we’ve discussed? |
| --- | --- |
| - Is there anything else you want to tell me? Do you have any questions for me? | |

## Thank them for their participation
